# Should Renal Inflammation Be Targeted While Treating Hypertension?

**DOI:** 10.3389/fphys.2022.886779

**Published:** 2022-06-13

**Authors:** Sarika Chaudhari, Grace S. Pham, Calvin D. Brooks, Viet Q. Dinh, Cassandra M. Young-Stubbs, Caroline G. Shimoura, Keisa W. Mathis

**Affiliations:** Department of Physiology and Anatomy, University of North Texas Health Science Center, Fort Worth, TX, United States

**Keywords:** resistant hypertension, blood pressure, immune cells, kidney, autoimmunity, systemic lupus erythematosus, lupus

## Abstract

Despite extensive research and a plethora of therapeutic options, hypertension continues to be a global burden. Understanding of the pathological roles of known and underexplored cellular and molecular pathways in the development and maintenance of hypertension is critical to advance the field. Immune system overactivation and inflammation in the kidneys are proposed alternative mechanisms of hypertension, and resistant hypertension. Consideration of the pathophysiology of hypertension in chronic inflammatory conditions such as autoimmune diseases, in which patients present with autoimmune-mediated kidney inflammation as well as hypertension, may reveal possible contributors and novel therapeutic targets. In this review, we 1) summarize current therapies used to control blood pressure and their known effects on inflammation; 2) provide evidence on the need to target renal inflammation, specifically, and especially when first-line and combinatory treatment efforts fail; and 3) discuss the efficacy of therapies used to treat autoimmune diseases with a hypertension/renal component. We aim to elucidate the potential of targeting renal inflammation in certain subsets of patients resistant to current therapies.

## Introduction

Hypertension remains the leading modifiable risk factor for cardiovascular morbidity, kidney disease, stroke, and premature death. In the last 3 decades, the global economic burden of hypertension has doubled not only due to a growing and aging population (Zhou et al., 2021), but also due to stress and a societal shift towards unhealthy lifestyles and diets. The International Society of Hypertension released new global practice guidelines in 2020, providing an updated definition of hypertension (Unger et al., 2020). The Society defined hypertension as a provider-measured blood pressure greater than 140/90 mm Hg, but recognized that 24-h ambulatory blood pressure monitoring in these individuals may show an average of greater than 130/80 mm Hg (Unger et al., 2020). The World Health Organization’s *Guideline for the Pharmacological Treatment of Hypertension in Adults* stresses the importance of having multiple blood pressure measurements, preferably across multiple visits, to accurately diagnose hypertension, but they recognize these conditions are not feasible in all settings (WHO, 2021). The United States continues to use hypertension guidelines set forth by the American Heart Association and American College of Cardiology in 2017 (Carey et al., 2018b). These guidelines define hypertension as blood pressure equal to or greater than 130/80 mm Hg, so that more efforts can be directed to control elevations in blood pressure before the disease progresses (Carey et al., 2018b). In the United States itself, half of the population has hypertension and only one out of four adults with hypertension have their blood pressure controlled (CDC, 2019b). Hypertension as a primary or contributing cause resulted in over 500,000 deaths in the United States in 2019 (CDC, 2019a).

Despite several advances in science, the etiology of hypertension still eludes physiologists (Ferdinand and Nasser, 2017). There are several mechanisms in place in the body to ensure blood pressure remains within physiological norms. Baroreceptors sense changes in blood pressure and then correct these changes through neural and hormonal pathways. These pathways augment heart rate, stroke volume, and total peripheral resistance, as well as control body fluid and electrolyte homeostasis (Pappano, 2010). The kidneys also have an undeniably important role in the fluid-electrolyte balance in the body through their intrinsic regulation of glomerular filtration rate and changes in electrolyte handling caused by neuroendocrine factors such as antidiuretic hormone, aldosterone, angiotensin II (ANGII), atrial natriuretic peptide, and brain natriuretic peptide (Oparil et al., 2018). Any disturbance of fluid-electrolyte homeostasis can contribute to hypertension (Coleman et al., 1975). As such, targeting water and electrolyte homeostatic systems through the kidney has been a cornerstone in the treatment of hypertension, although with limited success in some subpopulations of hypertensive patients.

It is well accepted that inflammation plays an important role in the pathogenesis of hypertension (Drummond et al., 2019). Although the role of the immune cells in hypertension has been known for decades (Okuda and Grollman, 1967; Svendsen, 1976), the direct role of inflammatory processes in the pathogenesis of hypertension was only recently appreciated. The causal role of lymph nodes and the thymus in hypertension in rodents was demonstrated in the 1960-70s (Okuda and Grollman, 1967; Svendsen, 1976). It has been confirmed that adaptive immunity with T lymphocytes [e.g., T helper (T_h_) cells] is an important player in modulating the inflammatory cytokine/chemokine production and hence in the immune-mediated pathogenesis of experimental hypertension in rodents (Guzik et al., 2007). Furthermore, knockout models such as B cell activating factor (BAFF) receptor knockout (BAFF-R^−/−^) mice or depletion of B and/or T cell activity using treatments like rituximab or mycophenolate mofetil blunt experimental hypertension in rodents (Herrera et al., 2006; Zheng et al., 2010; Chan et al., 2015; Taylor and Ryan, 2017; Taylor et al., 2018). The potential of mycophenolate mofetil to reduce blood pressure in chronic inflammatory settings has also been realized in hypertensive humans with psoriasis and rheumatoid arthritis (Herrera et al., 2006).

Cells of the innate immune system, including monocytes, macrophages, natural killer cells and dendritic cells (DCs) also contribute to hypertension (Lu and Crowley, 2020). Innate immunity is a non-specific response to antigens and causes inflammation directly through the activation of pattern recognition receptors such as toll-like receptors (TLRs) and nucleotide-binding oligomerization domain (NOD)-like receptors. Activation of these receptors on innate immune cells leads to increased oxidative stress while also increasing the release of various inflammatory cytokines that play important role in the development of hypertension (Mian et al., 2014; Van Beusecum et al., 2021). Indirectly they also cause activation of the adaptive immunity via the T_h_1 response or the humoral/antibody T_h_2 response, triggering further secretion of potent pro-inflammatory cytokines like interleukin (IL)-6, interferon (IFN)-γ, and IL-17, in turn contributing to the hypertension pathology (Mikolajczyk and Guzik, 2019).

Interestingly, several pharmacologic agents that reduce blood pressure have general anti-inflammatory effects as well (Silva et al., 2019). Below we discuss common therapies for hypertension and how hypertensive drugs from different classes are often used in combination to reach the target blood pressure reduction in situations where blood pressure is more difficult to control (Tsioufis and Thomopoulos, 2017). A complete list of pharmacologic anti-hypertensive therapies are summarized in Table 1. We will elaborate on those showing effects on the inflammation specifically.

**TABLE 1:** Common hypertensive drugs and their mechanisms of action.

Medication class	Example drug	Target/Mechanism of action	References
Renin inhibitors	Aliskiren	Decrease renin activity consequently decreasing ANG II	(Sears, 2008; WHO, 2021)
		Contraindicated in patients with diabetes	
Angiotensin-converting enzyme inhibitors	Captopril	Inhibits the conversion of ANG I to ANG II	Ram, (2008)
Angiotensin receptor blockers	Losartan	AT1 receptor blocker	Zhang et al. (2003)
Diuretics	Chlorothiazide	Increase urine excretion, lowering blood volume	Oparil, (2003)
		Side effect: Electrolyte imbalances	
Beta blockers	Propranolol	Reduce heart rate and contractility	(Shand, 1975; WHO, 2021)
		Preferred hypertension treatment in patients with heart failure	
Calcium channel blockers	Diltiazem	Relax vascular smooth muscle, lowers heart rate and contractility	Cushman et al. (2000)
		Common first-line therapy for people of African descent: more efficacious in this population	
Alpha blockers	Terazosin	Vascular relaxation, lowers total peripheral resistance	Achari and Laddu, (1992)
Central Alpha-2 receptor agonists	Methyldopa	Activates α_2_ receptors, providing negative feedback to reduce norepinephrine release	(Frohlich, 1980; WHO, 2021)
		Primary antihypertensive medication given during pregnancy	
Peripheral adrenergic inhibitors	Reserpine	Prevents the release of norepinephrine	Magarian, (1992)
		Prescribed when other medications do not work since it has more side effects	
Vasodilators	Hydralazine	Vasodilation	Kandler et al. (2011)
Endothelin receptor antagonists	BQ123	Prevents endothelin-1 mediated vasoconstriction and changes in sodium handling	(Moore and Linas, 2010; Kohan and Barton, 2014)
		Major side effect: edema	

Table 1 ANG I—angiotensin I; ANG II—angiotensin II.

### Blood Pressure-lowering Medications and Their Effect on Inflammation

The renin angiotensin system (RAS) is a target for common first-line therapies to treat hypertension (WHO, 2021). Renin is a peptide released by juxtaglomerular (JG) cells, modified smooth muscle cells of the afferent arteriole of the kidney nephron, when 1) perfusion pressure within the afferent arteriole decreases; 2) renal sympathetic nerves activate the smooth muscle in the afferent arterioles; or 3) the macula densa senses a decrease in NaCl concentration in the ultrafiltrate. Angiotensinogen, a peptide produced by the liver, is cleaved by renin to produce angiotensin I (ANG I). Angiotensin-converting enzyme (ACE), mostly located on pulmonary vascular endothelial cells, then further converts ANG I into the active form, ANG II (Pappano, 2010). ANG II acts on cells in the adrenal cortex and posterior pituitary to stimulate the release of aldosterone and ADH, respectively. These hormones, in concert with ANG II, promote vasoconstriction and increase sodium and water reabsorption in the kidney, effects that combine to increase blood pressure (Stanton and Koeppen, 2010). ANG II has also been implicated as a crucial mediator of immune and inflammatory processes in hypertension (Ruiz-Ortega et al., 2000; Benigni et al., 2010) and hence ANG II infusion is a popular and consistent *in vivo* animal model for inducing hypertension with an inflammatory component in preclinical research (Schiffrin and Touyz, 2003). Upon binding to its ANG II-type 1 (AT1) receptor, ANG II increases vascular permeability and increases chemokine expression, which aids the inflammatory process and promotes immune cell infiltration (Suzuki et al., 2003). AT1 activation also promotes the proliferation and activation of T_h_1 and T_h_17 cells, which are both pro-inflammatory (Liu et al., 2009; Platten et al., 2009). On the other hand, ANG II binding to its AT2 receptor may have anti-inflammatory effects (Benndorf et al., 2009).

Interventions blocking components of the RAS are effective in decreasing blood pressure since they act on several targets including the kidneys, vasculature, brain, and immune system. Inhibition of renin and ACE prevents downstream production and action of ANG II, limiting rises in blood pressure. Aliskiren is a renin inhibitor that was approved for the treatment of essential hypertension (Sears, 2008), but the benefits of this drug may span beyond blood pressure control as aliskiren also decreases levels of systemic inflammatory cytokines like CD45 and CD3 T cells as well as F4/80-positive macrophages in hypertensive mice (Yen et al., 2013). Captopril is an ACE inhibitor that effectively lowers blood pressure in humans and animals, but also has an anti-inflammatory effect (Fukuzawa et al., 1997; De Albuquerque et al., 2004; Sepehri et al., 2016; Gan et al., 2018; Mitchell et al., 2021). Another ACE inhibitor, lisinopril, was shown to reduce autoimmune-induced inflammation in a mouse model of multiple sclerosis (Platten et al., 2009). ANG II receptor blockers (ARB), such as losartan, prevent the binding of ANG II to the AT1 receptor, the receptor primarily responsible for the vasoconstrictor and pro-inflammatory effects of ANG II (Brasier et al., 2002; Zhang et al., 2003; Gonçalves et al., 2004). ARBs have been shown to be as effective as ACE inhibitors in reducing cardiovascular risks (Li et al., 2014) while also reducing systemic inflammation in various disease states (Fairbrass et al., 2021; Gamboa et al., 2012).

While agents targeting RAS are first-line drugs to defend against hypertension, there are other drugs that target different mechanisms. Diuretics like thiazides, potassium sparing diuretics, and loop diuretics are often used in combination with other antihypertensive drugs; they reduce blood pressure by increasing excretion of sodium and water. The loop diuretic, furosemide, reduced the concentrations of proinflammatory cytokines TNF-α, IL-6 and IL-1β and activated polarization of macrophages from proinflammatory M1 type to anti-inflammatory M2 type. (Yuengsrigul et al., 1999; Xu et al., 2006; Wang et al., 2020; Tuttolomondo et al., 2021). In addition, hydrochlorothiazide from the thiazide diuretic group inhibits the secretion of proinflammatory cytokines like TNF-α and IFN-γ (Luo et al., 2011; Aloud et al., 2020). Interestingly, the last decade has also emphasized the anti-inflammatory properties of calcium channel blockers in addition to their antihypertensive effects due to reduced entry of calcium in the cardiac cells and smooth muscle cells of the vasculature in turn decreasing the contractile force. The calcium channel blocker lercanidipine lowers the number of polymorphonuclear leukocytes and C reactive protein in patients of essential hypertension while nicardipine inhibits the Th2-mediated airway inflammation and IFN-γ-induced neuro-inflammation of the microglial cells. (Gomes et al., 2007; Farah et al., 2013; Huang et al., 2014; Saddala et al., 2020). Beta-blockers inhibit the action of norepinephrine and epinephrine on β-adrenergic receptors. These are generally used to reduce the work output of the heart, promote relaxation of the vasculature and lower the blood pressure (Shand, 1975). In addition, β-blockers inhibit renin release from the kidneys and have demonstrated anti-inflammatory cytokine profile after their use (Ohtsuka et al., 2001; Gage et al., 2004; Hagiwara et al., 2009; Jachs et al., 2021). Methyldopa is a centrally-acting α2-adrenergic receptor agonist (Frohlich, 1980) and analog of DOPA (3,4-hydroxyphenylanine) that inhibits the adrenergic neuronal outflow and vasoconstriction response reducing the blood pressure. Methyldopa blocked the antigen presentation and inflammatory T cell responses in type 1 diabetes patients, suggesting its potential to combat autoimmunity (Ostrov et al., 2018; Bogacz et al., 2021). The vasodilator class of anti-hypertensive drugs, for example hydralazine, is beneficial as an antioxidant and anti-inflammatory agent in rodent models of sepsis, renal and myocardial ischemia-reperfusion injury, and the spontaneously hypertensive rat (Rodrigues et al., 2008; Li et al., 2020; Leu et al., 2021; Santos et al., 2021). Endothelin-1 (ET-1) is a potent vasoconstrictor and regulator of sodium excretion that induces inflammation and oxidative stress, so endothelin receptor antagonists have been investigated as a treatment for hypertension (Kohan and Barton, 2014). These drugs have not had wide attraction or success in treating hypertension due to adverse side effects like significant edema (Moore and Linas, 2010; Kohan and Barton, 2014). It is not yet known if drugs from other classes of antihypertensives like peripheral α-1 adrenergic inhibitors have anti-inflammatory properties.

These studies linking the anti-inflammatory properties to anti-hypertensive medications do not necessarily indicate a causal role of inflammation in the pathogenesis of hypertension. An interesting study by Marvar et al. actually demonstrated that hydralazine lowered blood pressure and, in turn, indirectly prevented the ANG II-induced T cell activation and vascular infiltration of leukocytes (Marvar et al., 2010). The association between inflammation and antihypertensive drugs may vary depending on the type of the inflammatory marker studied, the disease model under investigation, and the stage of the disease. It is highly likely that inflammation and blood pressure modulation may influence each other in a complex way. For example the drug Etanercept, which blocks TNF-α, reduced blood pressure in ANG II-induced hypertension (Guzik et al., 2007) and an autoimmune model of chronic inflammation (Venegas-Pont et al., 2010), but not in salt-dependent hypertension (Elmarakby et al., 2008). The recent Canakinumab Anti-inflammatory Thrombosis Outcomes Study (CANTOS) demonstrated that the IL-1β inhibition with canakinumab reduced major adverse cardiovascular event rates and C reactive protein, without effecting blood pressure (Dixon et al., 2020). Nevertheless, these studies emphasize the importance of identifying subpopulations of patients that may respond to particular anti-inflammatory therapy, while another cohort may not respond to that same therapy.

The American College of Cardiology stresses the importance of lifestyle modification in addition to or in place of pharmacological agents (Goetsch et al., 2021). The World Health Organization suggests initial treatment with one drug targeting the RAS combined with a thiazide-like diuretic, along with a drug from a third category like calcium channel blockers if necessary (WHO, 2021). However, some medications may be preferred or contraindicated based on the patient’s situation, e.g., β-blockers are a preferred hypertension treatment for heart failure patients due to their beneficial effects on the heart, and certain RAS inhibitors are contraindicated for people of African descent, making calcium channel blockers a common first-line therapy for these patients (Unger et al., 2020). The most commonly used initial combination of therapies include a long-acting calcium channel blocker (CCB), a blocker of RAS (ACE inhibitor or ARB), and a diuretic. In many cases, it is still difficult to control blood pressure even with a combination of drugs. The American College of Cardiology and American Heart Association defines “resistant hypertension” as occurring when blood pressure remains above the target blood pressure of 130/80 mmHg despite concurrent use of three antihypertensive agents of different classes, or when four or more antihypertensive agents are needed to control blood pressure at the optimum level (Carey et al., 2018a). True resistant hypertension can be differentiated from the pseudo- or apparent resistant hypertension, in which the latter is due to measurement error, the “white coat effect”, or nonadherence to treatment. Use of proper blood pressure measurement techniques by trained staff, using out–of-office, ambulatory and self-monitoring of the blood pressure, monitoring prescription refills and achieving a good doctor-patient relationship are some of the ways to avoid pseudo-resistant hypertension (Carey et al., 2018a). The cause of resistant hypertension is typically further investigated for 1) suboptimal lifestyle factors, e.g., obesity, dietary sodium intake, and physical inactivity; 2) other drugs interfering with the action of the antihypertensive therapy; and/or 3) other conditions that lead to hypertension such as endocrine disorders, renal disease, renal artery stenosis, and coarctation of aorta (Carey et al., 2018a). The fact that resistant hypertension cannot be controlled easily by the current anti-hypertensive regimens calls for new cellular and molecular targets.

### The Role of Renal Inflammation in Hypertension

Many investigators have demonstrated the causal role of renal inflammation in the form of immune cell (both innate and adaptive) infiltration and a pro-inflammatory milieu in the pathogenesis of hypertension (Vanegas et al., 2005; Franco et al., 2006). In fact, recent studies demonstrate that the immune cell infiltration in the kidneys may alter the renal vascular function and the fluid electrolyte balance in addition to causing renal injury (Franco et al., 2013; Rucker et al., 2018). Infiltrating T cells in the kidneys accentuate the Dahl salt-sensitive phenotype by increasing intrarenal ANG II and oxidative stress (Mattson, 2014). Proinflammatory cytokines like IL-17A and IFN-γ can induce hypertension by altering the reabsorption of sodium through its transporters: sodium-hydrogen exchanger-3 (NHE3), sodium-potassium-chloride cotransporter (NKCC) and sodium-chloride cotransporter (NCC) on the renal tubules (Kamat et al., 2015). Additionally, the polarization of macrophages to the pro-inflammatory M1 phenotype in the kidney can promote macrophage recruitment and added release of various inflammatory cytokines leading to renal damage and altered fluid-electrolyte balance (Fehrenbach and Mattson, 2020). Studies show that stimulation of dendritic cells (DCs) in the kidneys results in oxidative stress, fluid retention, and increased blood pressure (Lu et al., 2020). The importance of DCs in hypertension was demonstrated by adoptive transfer experiments of splenic DCs from ANG II hypertensive mice that was able to activate T-cells in the recipient mice. Furthermore, adoptive transfer of DCs from animals that underwent renal denervation led to a decrease in total leukocytes, CD3^+^, CD4^+^ and CD8^+^ T cells in response to low dose of ANG II. This suggests an association of the renal sympathetic nerves and renal inflammation in the development of hypertension (Xiao et al., 2015), which has been confirmed by many others (Xiao et al., 2015; Banek et al., 2018; Banek et al., 2019; Osborn et al., 2021). Besides renal nerves, sympathoexcitation of other nerves also contributes to hypertension and the contribution of the sympathetic vasomotor outflow seems to be dependent on the hypertension model and the stage of the disease. For example, in ANG II-induced hypertension, denervation of the splanchnic nerve, but not the renal or lumbar nerve, was able to blunt hypertension (Osborn and Fink, 2010). On the other hand, both renal and splanchnic nerve contributes to hypertension in the Genetically Hypertensive Schlager (BPH/2J) mice (Asirvatham-Jeyaraj et al., 2021). Renal denervation was unable to reduce blood pressure in hypertensive mice with late-stage systemic lupus erythematosus (Mathis et al., 2013). Thus, sympathetic activation has an important, but complicated role in the development and maintenance of hypertension and renal inflammation and needs further investigation.

### Autoimmune-Induced Renal Inflammation Promotes Hypertension

Autoimmune diseases are characteristic of overactivation of immune cells, and chronic inflammation. Coincidently, autoimmune diseases with a renal component are associated with hypertension and cardiovascular morbidities (Boesen and Kakalij, 2021). Examples include systemic lupus erythematosus (SLE), Goodpasture syndrome, idiopathic membranous nephropathy, immunoglobulin A nephropathy, anti-neutrophil cytoplasmic antibody-associated glomerulonephritis, idiopathic thrombocytopenic purpura, rheumatoid arthritis and psoriasis (Panoulas et al., 2007; Coumbe et al., 2014; Giannelou and Mavragani, 2017; Liu and Kaplan, 2018; Tumurkhuu et al., 2019; Boesen and Kakalij, 2021). The renal pathological mechanisms involved in autoimmune-induced hypertension are still not completely understood; however, we do know that the loss of self-tolerance leads to autoantibody production and these autoantibodies form complexes, deposit into tissues like the kidneys and promote activation of other immune cells and the complement system. The resultant secretion of inflammatory mediators locally promotes chronic renal inflammation and oxidative stress (Choi et al., 2012; Small et al., 2018), which may increase fluid and electrolyte imbalance in the kidneys and/or cause renal vascular dysfunction leading to hypertension (Makino et al., 2002; Liu et al., 2012; Cowley et al., 2015; Gonzalez-Vicente et al., 2019).

SLE is the prototypical autoimmune disease with a renal inflammatory component usually referred to as lupus nephritis. Lupus nephritis develops following the production of diverse complex-forming nuclear autoantibodies that attack various self-antigens, including double-stranded DNA (dsDNA), histones, cardiolipins, phospholipids, Smith antigen and ribonucleoproteins (sm/RNP), and complement 1q (C1q) (Artim-Esen et al., 2014; Dema and Charles, 2016; Chen and Tsokos, 2021). Lupus nephritis is prevalent in more than half of SLE patients and it causes severe damage to glomerular, tubular and/or renal vascular structures (Najafi et al., 2001; Hsieh et al., 2011; Tsumiyama et al., 2013; Suarez-Fueyo et al., 2017; Ding et al., 2018). Lupus nephritis precedes hypertension in both female human SLE and the well-accepted female mouse model of SLE, the *NZBWF1* mouse, so this enables the investigation of contributory mechanisms of hypertension in the setting of chronic renal inflammation. In addition, therapies that suppress lupus nephritis warrant identification and introduction to the hypertension field so that the scope of use of these drugs in patients with hard-to-control hypertension with a renal inflammatory component (e.g., resistant hypertension) can be considered.

Currently the prevalence of SLE in the United States is 72.8 per 100,000, with a threefold increase in incidence due to improved screening measures (Izmirly et al., 2021), and 9 out of 10 SLE patients are women of reproductive age. There are extreme disparities in SLE, since Black, Hispanic, Asian and Native American women are most commonly affected for unknown reasons. Several studies have confirmed a high prevalence of hypertension in SLE patients, ranging from 40% to as high as 74% in some cohorts (Sabio et al., 2011). Hypertension is a prominent baseline risk factor for severe ischemic stroke and cardiovascular disease in SLE patients (Mikdashi et al., 2007; Sabio et al., 2011; Munguia-Realpozo et al., 2019). Since cardiovascular disease is the leading cause of mortality among SLE patients, it is critical that we understand the pathogenesis of hypertension in SLE.

Current guidelines disregard the specific management of hypertension in SLE patients and focus on the treatment of lupus nephritis, skin manifestations, and pulmonary hypertension instead. Tselios et al. does recommend, however, a reduction of blood pressure to ≤130/80 for SLE patients using ACE inhibitors and ARBs, a similar goal and regimen recommended by the American Society of Cardiology for hypertensive persons (Tselios et al., 2020). The use of ACE inhibitors like captopril may be beneficial since it reduces renal injury and inflammation in spontaneously hypertensive rats (SHR) while reducing blood pressure (Gan et al., 2018). Captopril also provides renal protection when administered to diabetic patients and Albuquerque et al. confirmed the renoprotective effects of captopril in a mouse model of autoimmunity (De Albuquerque et al., 2004). The same group discovered that ACE inhibition decreases the renal expression of the pro-inflammatory mediator transforming growth factor (TGF)-β. These studies stress captopril’s ability to improve renal inflammatory processes, but more studies are needed to determine the efficacy in SLE.

Evidence of the effect of first-line SLE therapies on blood pressure is limited. However, we do know that traditional therapies that target lupus nephritis possess various adverse effects (see Table 2). Corticosteroids, the most widely-used agent for all autoimmune diseases, cause systemic side effects such as psychosis, Cushing’s syndrome, and importantly, hypertension in SLE patients (Petri et al., 2014; Kassel and Odum, 2015), while the *NZBWF1* mice develop osteopenia and adrenal gland atrophy following chronic corticosteroid therapy (Jia et al., 2018). Novel immunomodulatory drugs are an exciting option for limiting immune system activation in SLE, but they often dampen the immune system excessively. For example, belimumab, a drug approved for SLE to inhibit B lymphocyte stimulator (BLyS) to induce apoptosis of autoreactive B cells (Weide et al., 2003; Petri, 2004; Pisoni et al., 2005; Chugh and Kalra, 2013), unfortunately causes progressive multifocal leukoencephalopathy in elderly SLE patients (Fredericks et al., 2014). Previous studies have shown that B cell or plasma cell depletion can prevent the development of hypertension if administered before the onset of SLE (Mathis et al., 2014; Taylor et al., 2018). Similar treatments exist for human SLE patients; rituximab binds to CD20 to deplete B cell activity, while mycophenolate mofetil and cyclophosphamide inhibit B cell activation and plasma cell synthesis. All have been efficacious in treating human SLE hypertension (Jiang et al., 2020; Lee et al., 2021). Mycophenolate mofetil also potentially reduces renal lymphocyte and macrophage infiltration while combating renal inflammation (Bravo et al., 2007). The most recent FDA-approved drug for SLE, anifrolumab, targets type 1 IFN by binding to its receptor IFNAR1 and inhibiting downstream inflammatory and immunological processes (Tanaka and Tummala, 2021). Anifrolumab caused adverse reactions such as nasopharyngitis, upper respiratory tract infection, and bronchitis (Mode and Stockholm, 2021), but its effect on renal inflammation and hypertension in SLE has not been investigated. Because of these unwanted and serious side effects, new and better therapies are still warranted to maintain the quality of life in SLE patients.

**TABLE 2:** A summary of drugs for SLE hypertension/lupus nephritis.

Medication class	Example drug	Target/Mechanism of action	References
Antimalarial	Hydroxychloroquine	Lysosomes, double-stranded DNA; inhibits immune activation and production of proinflammatory cytokines	Schrezenmeier and Dörner, (2020)
Corticosteroids	Methylprednisolone	Glucocorticoid receptors; inhibit many inflammation-associated molecules such as cytokines, chemokines, arachidonic acid metabolites, and adhesion molecules	Ocejo and Correa, (2021)
B cell inhibitors	Rituximab	CD20 on B cells; depletes B cell activity	de Bourcy et al. (2017)
	Mycophenolate mofetil	Inhibits B cell activation and plasma cell synthesis	Eickenberg et al. (2012)
	Cyclophosphamide	Inhibits B cell activation and plasma cell synthesis	Eickenberg et al. (2012)
	Belimumab	B lymphocyte stimulator inhibitor; inhibits B lymphocyte proliferation and differentiation into plasma cells, induces apoptosis of autoreactive B cells	Chugh and Kalra, (2013)
Type-I IFN inhibitor	Anifrolumab-fnia	Type I IFN Receptors; binds to IFNAR1, blocking action of all Type I IFNs, inhibiting downstream inflammatory and immunological processes	Tanaka and Tummala, (2021)
PPAR-γ agonist	Rosiglitazone	Adipose tissue; reduces ET-1, lowers blood pressure, reduces renal inflammation and injury	Venegas-Pont et al. (2009)
Protease inhibitor	Bortezomib	Chymotrypsin-like subunit of 26S proteasome; decreases production of autoantibodies and attenuates hypertension	Segarra et al. (2020)
Immunosuppressa-nts	Azathioprine	Incorporates into DNA and RNA to inhibit their synthesis, inhibits CD28-mediated signal in T cells	Fava and Petri, (2019)
	Methotrexate	Dihydrofolate reductase; interferes with DNA synthesis, repair, and replication, reducing purine synthesis, depletes folates	Fava and Petri, (2019)
Calcineurin inhibitor	Voclosporin	Binds and inhibits calcineurin, suppressing T cell activation and reducing renal inflammation	Rovin et al. (2021)

Table 2 DNA—deoxyribonucleic acid; RNA—-ribonucleic acid; IFN—interferon; IFNAR1—subunit one of the type 1 interferon receptor; PPARγ—peroxisome proliferator activator receptor-gamma; ET-1—Endothelin-1.

### Mechanisms That Regulate Renal Inflammation in SLE Hypertension

If we can target renal inflammation effectively, then there will likely be an improvement in blood pressure control in SLE given that majority of patients with lupus nephritis present with hypertension (Shaharir et al., 2015). Coincidently, SLE patients have significantly higher risks of resistant hypertension compared to non-SLE patients and this resistant hypertension is associated with lower renal function, and increased circulation inflammatory markers (erythrocyte sedimentation rate and C-reactive protein) (Gandelman et al., 2020).

**FIGURE 1:**
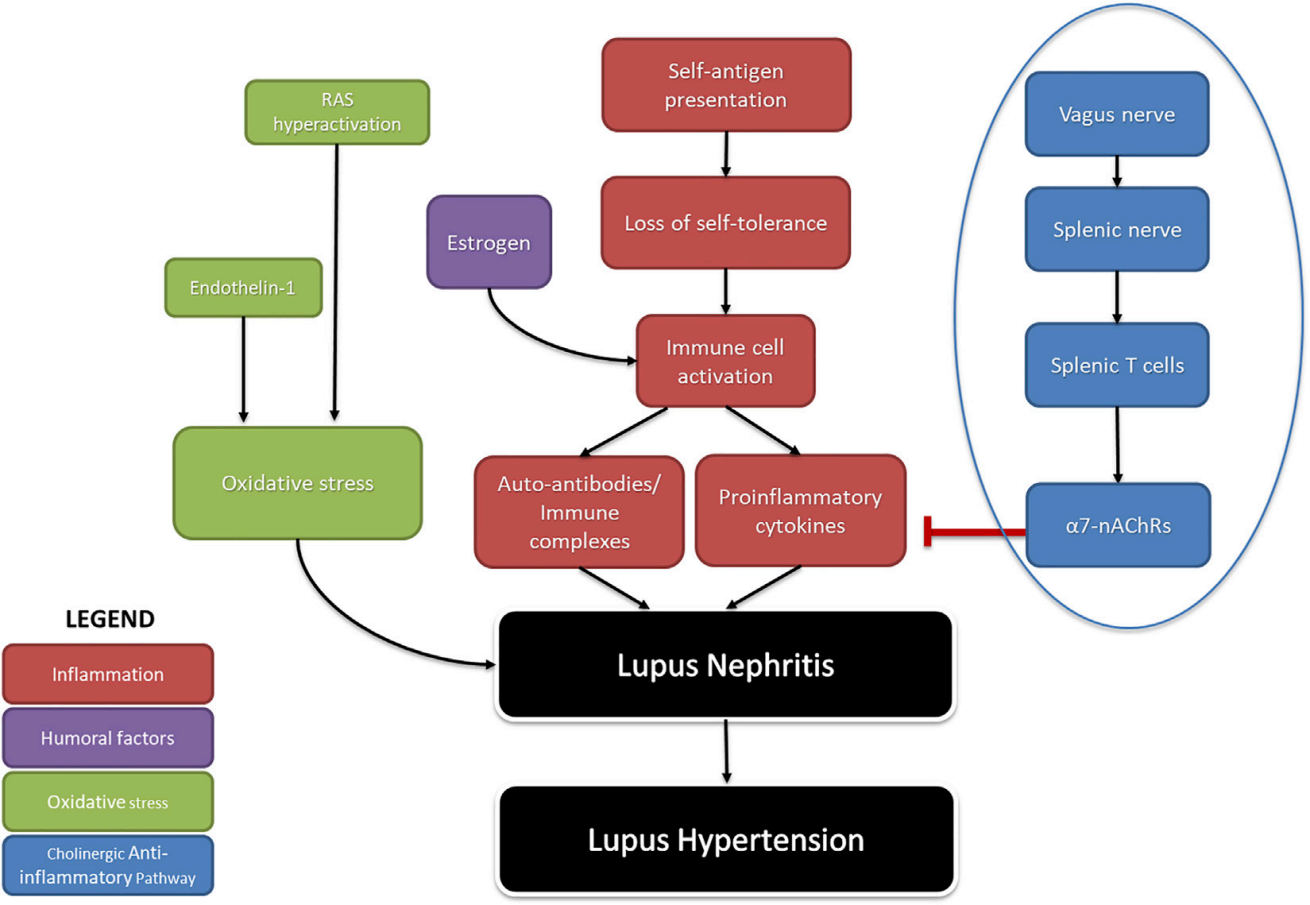
The pathophysiology of SLE-induced hypertension: A summary of factors that contribute to hypertension in SLE. Inflammation due to autoimmunity (red), as well as humoral factors that increase it (purple), and aberrant activity causing oxidative stress (green) comprise endogenous causes of lupus hypertension. The cholinergic anti-inflammatory pathway (blue) represents a neurogenic cause of lupus hypertension. Arrows (→) indicate stimulation; line with flathead (--|) indicates inhibition (RAS- renin angiotensin system; α7nAchRs-alpha seven nicotine acetyl choline receptors).

Many studies have revealed mechanistic pathways regulating renal inflammation in SLE hypertension (Figure 1) (Ryan, 2009). Because SLE is a female-dominant disease, we often turn to the contributions of estrogen. Estrogen stimulates autoreactive B cells to increase autoantibody production, resulting in the rise of pro-inflammatory cytokines such as tumor necrosis factor (TNF)-α, IL-6, and C-reactive protein. Pharmacological blockade of estrogen early in life reduces renal inflammation by decreasing the population of B cells and immune complex deposition in *NZBWF1* mice (Wu et al., 2000; Sthoeger et al., 2003). This suggests a mechanistic role of estrogen in contributing to renal inflammatory processes early in life in SLE. Another mechanism involves the increased production of endothelin-1, along with hyperactivation of the RAS increasing oxidative stress, which has been known to promote SLE hypertension (Kuryliszyn-Moskal et al., 2008; Mathis et al., 2012; Shah et al., 2014; Munguia-Realpozo et al., 2019). Since endothelin receptor A (ETA) blockade ameliorates renal inflammation and albuminuria in the diabetic rats (Saleh et al., 2011) it may be capable of doing the same in SLE hypertension.

**FIGURE 2:**
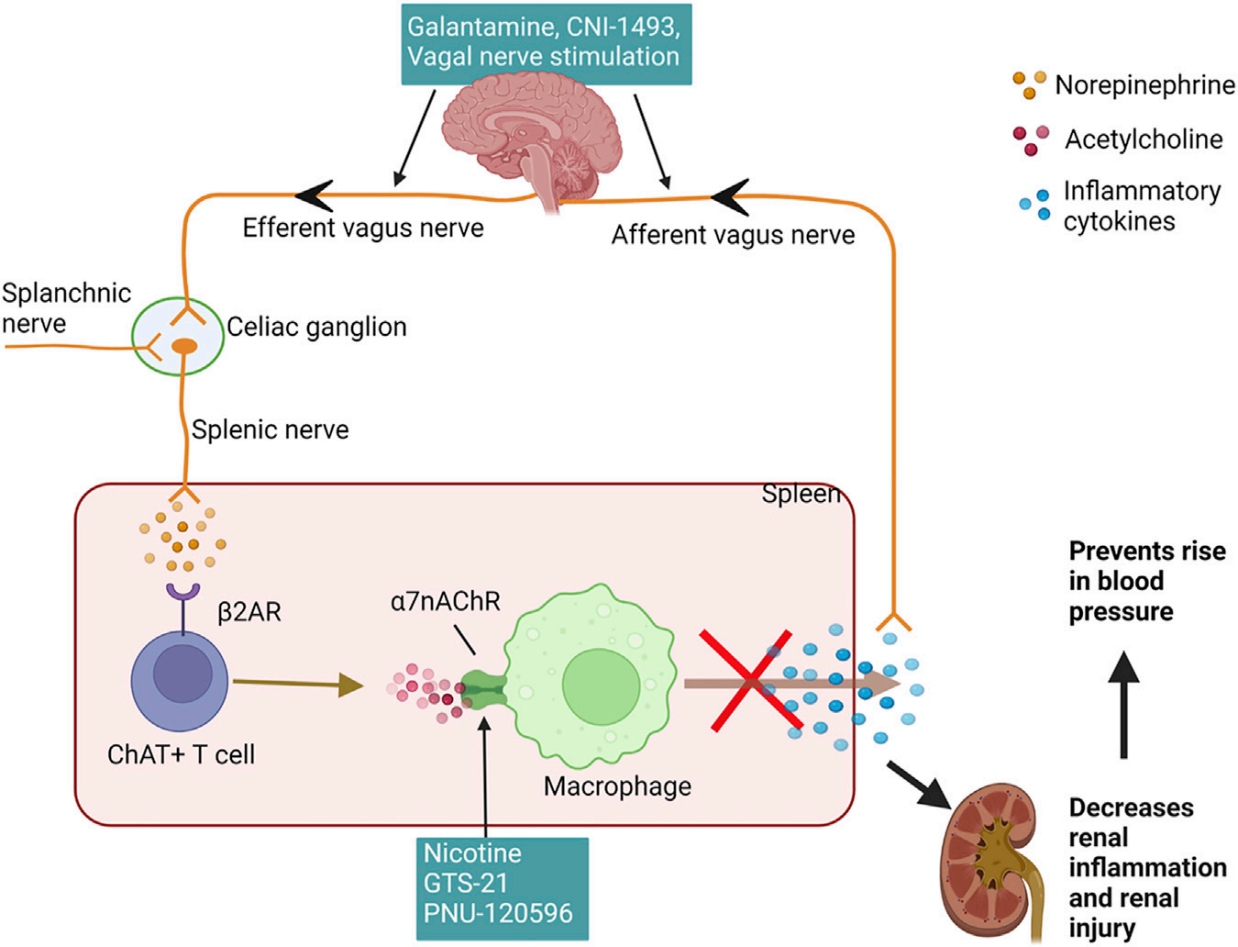
Cholinergic anti-inflammatory pathway reduces renal inflammation and hypertension: The afferent vagus nerve detects inflammatory cytokines and relays this information centrally to increase the efferent vagus, which synapses on the celiac ganglion and activates the splenic nerve. Another possible mechanism for activation of splenic nerve is via the splanchnic nerve. The splenic nerve has sympathetic fibers and stimulates splenic ChAT + T cells to synthesize and secrete acetylcholine, which acts upon various immune cells in the spleen, including macrophages, to inhibit the production and release of inflammatory cytokines. A reduction in splenic inflammation and proinflammatory cytokines in circulation decreases renal inflammation and renal injury preventing the rise in the blood pressure. The efferent vagus nerve can be stimulated using pharmacological agents galantamine and CNI-1493 or direct electrical stimulation. GTS-21 and nicotine, agonists of the α7nAchR and PNU-120596, positive allosteric modulator for this receptor leads to activation of this receptor to inhibit the cytokine release from immune cells. (β2AR—beta 2 adrenergic receptor; ChAT + T cells—choline acetyltransferase positive T cells; α7nAchRs—alpha seven nicotine acetylcholine receptors). Created with Biorender.com.

Less discussed are the possible neurogenic influences on renal inflammation in SLE hypertension. Basal activity of the hypothalamic-pituitary-adrenal (HPA) axis contributes to the suppression of peripheral inflammation by the release of corticosterone. Additionally, decreased parasympathetic (vagal) tone, as indicated by decreased heart rate variability, has been consistently identified in clinical studies involving SLE patients since 1997 (Laversuch et al., 1997; Maule et al., 1997; Thanou et al., 2016; Matusik et al., 2018; Zinglersen et al., 2021). An impaired HPA axis is present in SLE and may explain why renal inflammation is not resolved in SLE (Rovenský et al., 1998; Pham and Mathis, 2018). Tracey et al. established the significance of the inflammatory reflex—specifically the efferent arm of this reflex, known as the cholinergic anti-inflammatory pathway—a neural circuit that attenuates excessive inflammation (Huston et al., 2007; Tracey, 2021). This circuit runs from the vagus nerve to the splenic nerve and causes the release of acetylcholine from splenic T cells. This acetylcholine activates the alpha seven subunit of the nicotinic acetylcholine receptor (α7-nAChR) on splenic immune cells, ultimately resulting in the blunted release of proinflammatory cytokines and overall suppression of systemic inflammation and tissue damage (Pavlov et al., 2009; Martelli et al., 2014a); (Figure 2). Both the cholinergic anti-inflammatory pathway and the HPA axis rely on an active vagus nerve; therefore, decreased vagal nerve activity in SLE likely contributes to decreased activity of both of these anti-inflammatory pathways (Stein et al., 1996; Thanou, 2014; Mathis, 2015).

Studies from our lab suggest that stimulation of this cholinergic anti-inflammatory pathway can attenuate renal inflammation and the progression of hypertension in SLE, presenting the nerve as a novel, potential target for intervention for SLE hypertension (Fairley and Mathis, 2017). Galantamine is a centrally acting acetylcholinesterase inhibitor that enhances efferent vagus nerve activity and further enhances the cholinergic anti-inflammatory pathway (Pavlov et al., 2009), (Figure 2). Chronic systemic administration of galantamine in SLE mice successfully protects from hypertension by attenuating renal and splenic inflammation, while also decreasing the levels of autoantibodies (Pham et al., 2018). Similarly, the action of semapimod hydrochloride (CNI-1493), a tetravalent guanylhydrazone molecule, which inhibits the synthesis of proinflammatory cytokines, is mediated in part by the activation of efferent vagal nerve fibers (Borovikova et al., 2000; Bernik et al., 2002). When treated with systemic CNI-1493, SLE mice showed attenuated hypertension, decreased albuminuria, improved renal blood flow and decreased renal vascular resistance (Maloy et al., 2016). Whether long-term activation of the vagus nerve attenuates renal inflammation and hypertension in SLE patients is a prospective study to consider. Already, a randomized controlled pilot trial subjecting human SLE patients to 4 days of transcutaneous auricular vagus nerve stimulation successfully reduced pain and fatigue in these patients (Aranow et al., 2021), but the effect on renal inflammation and hypertension were not considered. Taken together, vagus nerve stimulation is promising in animal studies and humans so additional studies are needed to support these findings and to understand the detailed molecular mechanisms for its effect in the pathogenesis of lupus nephritis and SLE hypertension.

However, we must consider that the cholinergic anti-inflammatory pathway features several communication nodes, namely the vagus nerve, celiac ganglion, splenic nerve, and spleen (Tracey, 2007; Rosas-Ballina and Tracey, 2009; Olofsson et al., 2012; Pavlov and Tracey, 2017). Yet, this pathway and the proposed connections are controversial (Martelli et al., 2014a; Martelli et al., 2014c). Specifically, the neuroimmune mechanism is thought to be mediated by communication between the vagus and splenic nerves via the celiac ganglion. Several studies have shown the importance of these nodes of transmission in the protection offered by vagus nerve stimulation (Borovikova et al., 2000; Huston et al., 2008; Rosas-Ballina et al., 2008); however, morphological and anatomical studies suggest there may be no such connection between the two nerves (Martelli et al., 2014c), that the source of catecholamines may paradoxically be the vagus itself (Verlinden et al., 2016), and/or that other nerves like the splanchnic nerve may be the source of activation instead (Figure 2; Martelli et al., 2014b; Komegae et al., 2018). A recent study from our lab showed that unilateral vagotomy decrease renal inflammation and blunt hypertension in SLE mice, contrary from our initial hypothesis that vagotomy would worsen SLE hypertension and inflammation due to the disruption of the anticholinergic anti-inflammatory pathway. These data suggest the existence of compensatory neuroimmune mechanisms that prevail even after unilateral vagotomy that need further attention and investigation (Pham et al., 2020).

Attempts at reducing renal inflammation and attenuating SLE hypertension at other nodes within the cholinergic anti-inflammatory pathway were less successful in our hands. While systemic administration of nicotine, an agonist of α7-nAChR (Figure 2), reduced splenic and renal cortical expression of proinflammatory cytokines and blood pressure (Fairley and Mathis, 2017), partial agonist, GTS-21, and PNU-120596, a positive allosteric modulator (PAM) did not significantly reduce inflammation or blood pressure in SLE mice (Morales et al., 2021). However, it is possible that the lack of beneficial effect of GTS-21 and PNU-120596 was due to the mice being treated at an advanced stage of SLE, and future studies that use an earlier timeline for the administration of these α7 ligands may prove to be more successful. Additionally, the absence of a coexisting endogenous agonist (i.e., acetylcholine) due to decreased vagal tone in SLE may lead to ineffectiveness of PAM action. Interestingly, the importance of cholinergic anti-inflammatory pathway in the treatment of resistant hypertension has recently been recognized. In their study, Hilderman et al. collected whole blood samples at different time points from patients with resistant hypertension treated with renal denervation. These samples, when further treated with GTS-21, showed long-term suppression of LPS-induced TNF compared to the short-term suppression in the vehicle group. Although the authors could not study the effects on renal inflammation, their study and ours suggest a role of compromised anti-inflammatory pathways in the development of renal inflammation in SLE hypertension (Hilderman et al., 2019).

The antimalarial drug hydroxychloroquine, commonly used in SLE, suppresses the activity of lysosomal enzymes, thereby preventing major histocompatibility complex (MHC) class II-mediated autoantigen presentation. This drug also binds to dsDNA and inhibits toll-like receptor (TLR) signaling and/or TLR binding in order to reduce the production of proinflammatory cytokines (Rainsford et al., 2015). Gomez-Gutman *et al.* showed that hydroxychloroquine decreases renal inflammation, blood pressure, and renal injury, but does not decrease levels of anti-dsDNA antibodies in the *NZBWF1* mouse model (Gómez-Guzmán et al., 2014). However, hydroxychloroquine was administered to the *NZBWF1* mice at 30 weeks of age, when autoimmune-mediated hypertension and renal injury have already developed. It is likely that the timing of hydroxychloroquine administration is crucial to attenuate the extent of the autoantibodies production, reflected by the fact that hydroxychloroquine is the first-line drug for mild, early SLE in humans (Gies et al., 2020).

Finally, other drugs with unique mechanisms are also beneficial in reducing renal inflammation and blood pressure in SLE, introducing other potential mechanisms. Rosiglitazone, a peroxisome proliferator activator receptor gamma (PPARγ) agonist, targets adipose tissue and has been shown to reduce endothelin-1 and attenuate hypertension as well as renal inflammation and injury in SLE mice model (Venegas-Pont et al., 2009). The TNF-α inhibitor, etanercept, decreases mean arterial pressure as well as renal injury measured by glomerulosclerosis and albuminuria, and also decreases renal monocyte infiltration and oxidative stress (Venegas-Pont et al., 2010). Etanercept has been approved in many autoimmune conditions like rheumatoid arthritis, psoriasis, and systemic sclerosis to alleviate TNF-α-mediated immune responses and inflammation. It also decreases blood pressure in AngII-induced hypertension (Guzik et al., 2007) and pulmonary hypertension (Zhang et al., 2016); however, its safety and role in human SLE hypertension needs further investigation. Another promising drug for treating lupus nephritis is calcineurin inhibitors, drugs that block T cell activation through the suppression of the calcium/calcimodulin-dependent phosphatase. The novel calcineurin inhibitor, voclosporin acts by reducing the activation of T cells, causing stabilization of podocytes and reduction of proteinuria (Mok, 2017; Mejía-Vilet and Romero-Díaz, 2021). Voclosporin has recently been approved as induction therapy in lupus nephritis in combination with myocophenolate mofetil and a glucocorticoid. Its effects on blood pressure in lupus have not been demonstrated (Rovin et al., 2021).

In summary, several immune and molecular pathways play a role in executing the control of renal inflammation. Future mechanistic studies exploring the possible pathways that contribute to the renal inflammation in SLE-induced hypertension may help to modify the treatment strategies for hypertension in the setting of chronic inflammation or resistant hypertension.

## Conclusion

There have been tremendous advances in treating hypertension. However, there are still subpopulations of hypertensive patients that do not respond to current combinations of antihypertensive agents indicating that alternative mechanisms are at play. In this review, we have provided an overview of what is currently known of the association between inflammation and hypertension, and evidence of the need to target renal inflammation in some hypertension patients. We provide intriguing examples that suggest that hypertension therapy could be more efficacious in those with underlying renal inflammation if the therapy was combined with an agent that combats renal inflammatory processes. There is a high prevalence of hypertension in SLE, so this presents an important disease model to study the control of renal inflammation in hypertension. Resistant hypertension is highly prevalent in SLE patients, so the link between the two also warrants further investigation. Some common SLE therapies have been successful in reducing both renal inflammation and blood pressure, while the efficacies of others have not been determined in humans or animal models of SLE. Studies determining the ability of other common SLE drugs to reduce blood pressure in more conventional animal models of hypertension and in hypertensive humans are warranted. Indeed, McCarthy et al. has already demonstrated the beneficial effects of chloroquine in spontaneously hypertensive rat (McCarthy et al., 2016) and chloroquine is well known to decrease vascular resistance and blood pressure in humans (Anigbogu et al., 1993). Development of diagnostic tools that identify the cohorts of hypertensive patients responding to a particular anti-inflammatory therapy combating renal inflammation is a step further towards personalized medicine. Whether drugs used to treat SLE have a beneficial effect on hypertension in the vulnerable population of resistant hypertensive patients is intriguing and warrants investigation.
